# Detecting Ground Glass Opacity Features in Patients With Lung Cancer: Automated Extraction and Longitudinal Analysis via Deep Learning–Based Natural Language Processing

**DOI:** 10.2196/44537

**Published:** 2023-06-01

**Authors:** Kyeryoung Lee, Zongzhi Liu, Urmila Chandran, Iftekhar Kalsekar, Balaji Laxmanan, Mitchell K Higashi, Tomi Jun, Meng Ma, Minghao Li, Yun Mai, Christopher Gilman, Tongyu Wang, Lei Ai, Parag Aggarwal, Qi Pan, William Oh, Gustavo Stolovitzky, Eric Schadt, Xiaoyan Wang

**Affiliations:** 1 Sema4 Stamford, CT United States; 2 Lung Cancer Initiative Johnson & Johnson New Brunswick, NJ United States; 3 Icahn School of Medicine at Mount Sinai New York, NY United States

**Keywords:** natural language processing, ground glass opacity, real world data, radiology notes, longitudinal analysis, deep learning, bidirectional long short-term memory (Bi-LSTM), conditional random fields (CRF)

## Abstract

**Background:**

Ground-glass opacities (GGOs) appearing in computed tomography (CT) scans may indicate potential lung malignancy. Proper management of GGOs based on their features can prevent the development of lung cancer. Electronic health records are rich sources of information on GGO nodules and their granular features, but most of the valuable information is embedded in unstructured clinical notes.

**Objective:**

We aimed to develop, test, and validate a deep learning–based natural language processing (NLP) tool that automatically extracts GGO features to inform the longitudinal trajectory of GGO status from large-scale radiology notes.

**Methods:**

We developed a bidirectional long short-term memory with a conditional random field–based deep-learning NLP pipeline to extract GGO and granular features of GGO retrospectively from radiology notes of 13,216 lung cancer patients. We evaluated the pipeline with quality assessments and analyzed cohort characterization of the distribution of nodule features longitudinally to assess changes in size and solidity over time.

**Results:**

Our NLP pipeline built on the GGO ontology we developed achieved between 95% and 100% precision, 89% and 100% recall, and 92% and 100% *F*_1_-scores on different GGO features. We deployed this GGO NLP model to extract and structure comprehensive characteristics of GGOs from 29,496 radiology notes of 4521 lung cancer patients. Longitudinal analysis revealed that size increased in 16.8% (240/1424) of patients, decreased in 14.6% (208/1424), and remained unchanged in 68.5% (976/1424) in their last note compared to the first note. Among 1127 patients who had longitudinal radiology notes of GGO status, 815 (72.3%) were reported to have stable status, and 259 (23%) had increased/progressed status in the subsequent notes.

**Conclusions:**

Our deep learning–based NLP pipeline can automatically extract granular GGO features at scale from electronic health records when this information is documented in radiology notes and help inform the natural history of GGO. This will open the way for a new paradigm in lung cancer prevention and early detection.

## Introduction

The goal of lung cancer treatment is primary prevention, early prediction, and detection of lung malignancy to reduce lung cancer mortality. Currently, prevention screening programs have proven to be effective in the early detection of many cancers [1]. Low-dose computed tomography (CT) has been a standard method for lung cancer screening in the United States since the National Lung Screening Trial in 2011 [2,3]. With the increased utilization of CT scans and advances in CT techniques, the detection rate of pulmonary nodules has increased during the last decade [4]. Approximately 20% to 30% of CT images detect pulmonary nodules with ground-glass opacity (GGO), a subtype of pulmonary nodules [5-7]. GGOs, either pure GGOs (without a solid component) or part-solid GGOs (with a solid component), have gained significant attention in recent years due to their malignancy potential [8-11] ever since Jang and colleagues [12] found that ground-glass attenuation could be a sign of lung adenocarcinoma. However, identifying malignant lesions based on GGO images from CT scans remains a challenge since both benign and malignant lung lesions can appear as GGOs [13-15]. Persistent GGOs, which have not been resolved in subsequent CT scans between 6 and 12 months, are more likely to be associated with precancerous or cancerous conditions, while transient and self-resolving GGOs are benign [16-19]. Other GGO features such as larger baseline nodule size, spiculated shape, upper lobe location, presence of a solid component, and less than 5 nodules in quantity are known to be highly associated with the probability of malignancy [20-23]. Understanding the characteristics and prognosis of GGOs is critical for predicting and preventing lung cancer development by adopting proper management [24,25].

Radiomics is a study field leveraging artificial intelligence (AI) to extract medical information from radiology images. Recent advances in radiomics have significantly improved the accuracy of identifying malignant lesions [26-28] and made possible differentiating etiologies of GGOs [29]. However, limited access to scans, the high cost, and the complexity of processes [30-32] have hindered the routine knowledge extraction from CT scans and prompted the use of patient electronic health records (EHRs). EHRs are rich sources of patients’ clinical information including radiological findings [33,34], which are generally captured in unstructured data fields. However, large-scale extraction of GGO information from an enormous collection of unstructured EHR data is almost impossible without leveraging the power of natural language processing (NLP).

NLP is an AI approach that enables extracting large-scale information automatically from clinical notes and presenting the extracted information in a computer interoperable structured format. Over the last 2 decades, NLP has played a critical role in representing medical information that is embedded in unstructured clinical notes [35-39] and has been applied to the field of radiology [40]. Pons et al [34] systematically reviewed 67 NLP studies in radiology reports and demonstrated how radiology fields benefit from NLP techniques. Linna and Kahn [41] also highlighted the potential benefits of NLP technology in multiple areas, such as improved diagnostic decision-making, patient care, and delivery. Although the development of deep learning methods and transformer models like Bidirectional Encoder Representations From Transformers (BERT) showed a significantly improved impact in named entity recognition and relation extraction [42], these state-of-the-art NLP methods have not been applied yet to extract data on GGOs and their related features. A few shallow NLP parsers have been developed to identify cohorts with GGOs [14,43-46]. Recently, a rule-based GGO NLP algorithm was developed and applied in combination with negation and temporal algorithms to extract and characterize all GGO attributes from radiology reports [4].

This study aimed to investigate the feasibility of developing a deep learning–based NLP model to extract GGO features systematically from radiology notes for the longitudinal analysis of patient-level GGO features on a large scale with ontology-guided contextual embedding and temporal reasoning. The utility of the NLP was then evaluated by deploying it to longitudinal data to assess changes in GGO features longitudinally, which is vital for understanding the natural history of GGOs in the real-world lung cancer setting.

## Methods

### Ethics Approval

This study was approved by the Program for the Protection of Human Subjects at the Mount Sinai School of Medicine (IRB-17-01245).

### Study Cohort

**Figure 1:**
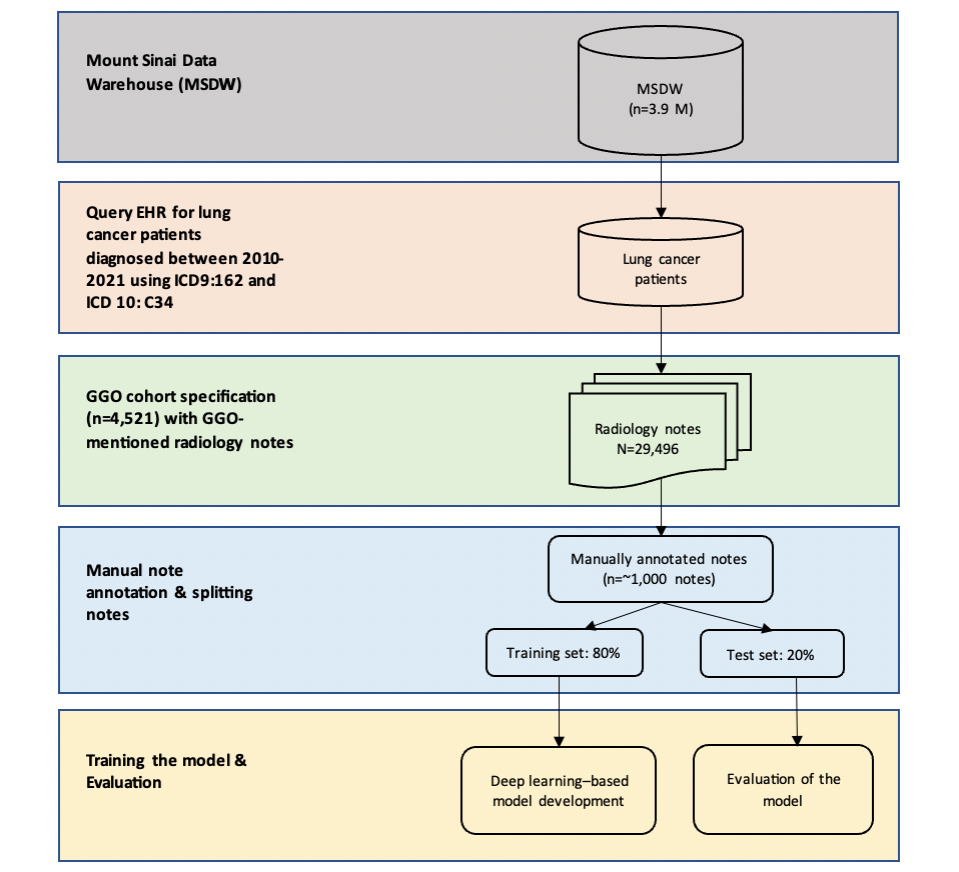
The workflow of the ground-glass opacity (GGO) natural language (NLP) pipeline. The workflow shows how we selected study cohorts and their radiology notes from EHRs for the next steps of model training and evaluation. EHR: electronic health record; ICD: International Classification of Diseases.

The cohort of patients diagnosed with lung cancer between 2010 and 2021 (13,216 patients) was curated from the Mount Sinai/Sema4 Healthcare system, which contains longitudinal data for approximately 3.9 million patients. Demographic and other clinical variables were obtained by either extracting from structured data or curating the relevant information from unstructured clinical notes (ie, radiology notes and progress notes). The study cohort includes (1) pathology-confirmed patients with lung cancer; (2) non–pathology-confirmed patients with lung cancer via ≥3 visits and International Classification of Diseases (ICD) lung cancer codes (ICD-9: 162 and ICD-10: C34); and (3) non–pathology-confirmed patients who had <3 visits with lung cancer ICD codes. We curated these initial lung cancer cohorts to develop and test the GGO NLP pipeline, which can then be applied to other relevant cohorts in the future. Figure 1 shows how we selected study cohorts and their radiology notes from EHRs for the next steps of model training and evaluation.

### NLP Framework

#### Overview

The framework we propose to curate GGOs and their related attributes are described as follows: (1) preprocessing and query expansion; (2) GGO ontology construction and annotation; (3) NLP model development; (4) postprocessing and entity normalization; and (5) NLP pipeline evaluation. These are discussed in greater detail in the following subsections.

#### Preprocessing and Query Expansion

The preprocessing phase focused on query expansion. An initial list of seed terms was obtained from a manual survey of the literature and a review of clinical notes by a clinical researcher and a domain expert (authors KL and MM). A bigram word2vec algorithm [47] was developed to identify additional significant terms potentially related to GGO to ensure the encapsulation of an expansive cohort. The expanded list of query terms was then applied to extract a comprehensive set of GGO-specific patient notes that were subsequently leveraged for NLP modeling.

#### GGO Ontology and Annotation

**Figure 2:**
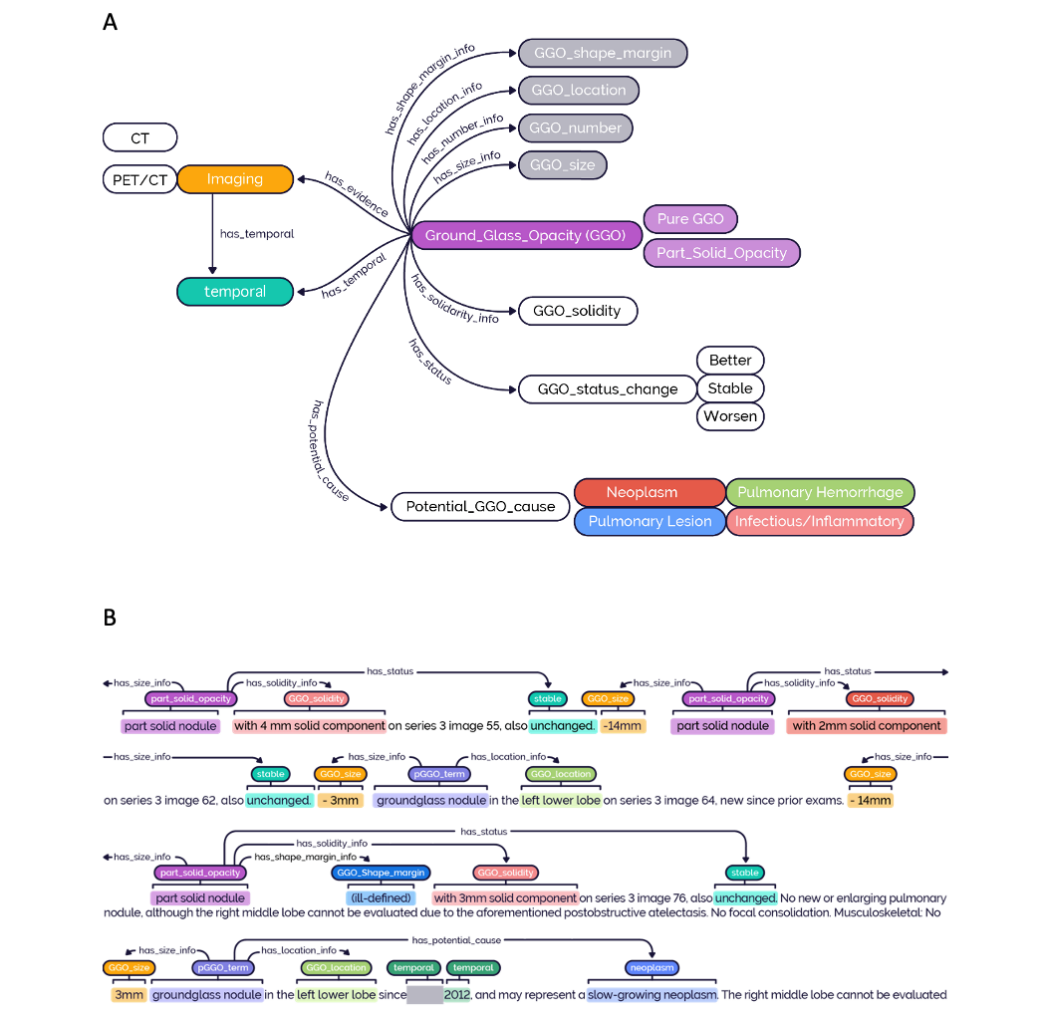
The ontology of ground-glass opacity (GGO) and the sample note with GGO annotations. A) The ontology of GGO. A total of 15 entities and 7 semantic relation types were defined in the GGO ontology. Entity semantic types: GGO location, GGO number, GGO shape/margin, GGO size, GGO solidarity, GGO status change: better, GGO status change: stable, GGO status change: worsen, GGO term: pure GGO term, GGO term: part-solid GGO, potential GGO cause: infectious/inflammatory, potential GGO cause: neoplasm, potential GGO cause: hemorrhage, potential GGO cause: other pulmonary lesions, and temporal. Relation semantic types: has location info, has number info, has shape/margin info, has size info, has solidarity info, has status, and has potential causes. B) Sample deidentified radiology reports with GGO annotations. Each part-solid nodule or ground-glass nodule is associated with attributes (such as size, location, status, change, shape, and/or solidity information) and potential etiologies. The upper panel shows a radiology report with multiple GGOs and their attributes; the lower panel shows a GGO and its associated potential etiologies. CT: computed tomography; PET: positron emission tomography.

NLP is the process of simulating an expert’s knowledge and understanding of the free text using modeling. As the first step of NLP, we built up an ontology that was established based on clinical expert opinion, comprehensive literature, and patient note review. The GGO ontology includes entities that are critical for cancer prediction based on previous studies and available from our radiology notes. Our GGO ontology includes 15 entities comprising pure GGO, part-solid GGO, GGO size, GGO quantity (number), GGO location, GGO shape/margin, GGO solidity, temporal (date), potential GGO cause (neoplasm, infectious/inflammation, hemorrhage, and other pulmonary lesions), and GGO status change (better, stable, and worsen). Moreover, it has 7 semantic relations between entities: has size information (info), has number info, has location info, has shape/margin info, has solidity info, has status, has a potential cause (Figure 2A). This ontology was used as a guideline for manual annotation. GGO status change indicates any description of size or solidity changes (eg, increased, getting smaller, getting denser). The primary GGO entities, either pure or part solid, were associated with their attributes like size, location, and so on. Then, 2 independent domain experts manually annotated the 15 entities and 7 semantic relations in the clinical notes (Figure 2B) using the Clinical Language Annotation, Modeling, and Processing (CLAMP) NLP toolkit [48], and a third domain expert (KL) reviewed the annotations.

Since a biomedical concept could be described in heterogeneous forms, continuous discussions and agreement between annotators and domain experts were needed to confirm that the annotations represented the expert’s understanding of biomedical knowledge. Interannotator agreement scores (kappa scores) were measured between the first 2 annotators in the same set of notes until they reached over 90% in entities and over 80% in relation annotation before commencing the independent annotation.

#### NLP Model Development

**Figure 3:**
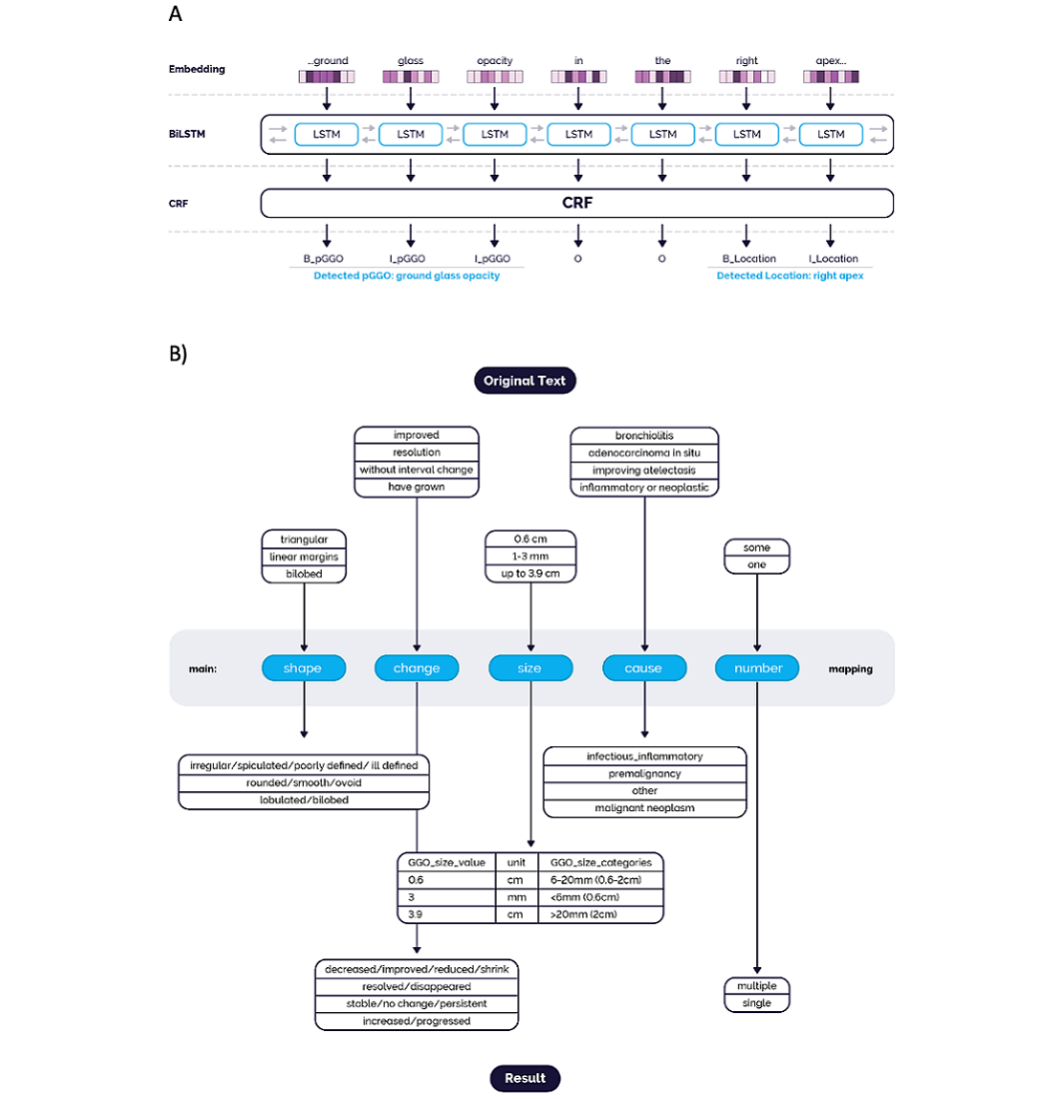
A deep learning natural language processing (NLP) pipeline for ground-glass opacity (GGO) curation and the process of GGO entity normalization. A) Multilayer deep learning NLP architecture for GGO curation. All clinical notes underwent word embedding before being sent to the bidirectional long-short term memory (Bi-LSTM), an artificial neural network of text classification architecture. The outputs were fed to a conditional random fields (CRF) model to predict the GGO entities and relations. B) GGO entity normalization. The raw outputs of NLP models (upper panel) were normalized to standardized concepts (lower panel) for each GGO attribute (middle panel).

A multilayer deep learning architecture was implemented for NLP modeling. The text was first transformed as sequential vectors of characterization in the embedding step. The vectors were then sent to the bidirectional long-short term memory (Bi-LSTM), an artificial neural network of text classification architecture, for pattern recognition in both forward and backward directions [49]. The patterns were sent to the next layer of a conditional random field (CRF) model to compute prediction probability (Figure 3A) [50]. In the example sentence of Figure 3A, the “ground-glass opacity” is predicated as the entities of “GGO,” while “right apex” is predicated as “location.” The model was trained, calibrated, and tested for optimal performance. Among manually annotated clinical notes, 80% (798/998) were used for training the GGO model and 20% (200/998) were used for validation.

#### Postprocessing and Entity Normalization

A postprocessor was developed to subsequently postcoordinate and refine the output. All predicated entities from the raw text were normalized to standardized concepts based on clinical experts’ opinions and were then ready for downstream analysis. Figure 3B illustrates examples of extracted GGO feature entities categorized and normalized for the data analysis. GGO location was extracted and classified into 2 levels; the first level corresponded to a high-level indication of right, left, or bilateral lungs, and the second level corresponded to a more granular indication of the anatomic location like right upper lobe (RUL), right middle lobe (RML), or right lower lobe (RLL), left upper lobe (LUL), and left lower lobe (LLL)*.* We categorized GGO size into 3 groups: <6 mm, 6 to 20 mm, and >20 mm based on expert opinion and the practice guidelines for nonsolid nodules. Potential etiologies found in the notes were classified into 3 subgroups: infectious/inflammatory, malignant, and others, whereby precancerous conditions such as atypical adenomatous hyperplasia and adenocarcinoma in situ were included in the malignant category. Others include all benign pulmonary lesions like fibrosis/scarring and hemorrhage.

#### NLP Pipeline Evaluation

The performance of the GGO NLP pipeline was estimated in the validation set with precision via the positive predictive value (PPV) and recall via sensitivity, as well as *F*_1_-score, a balanced score between false positives (FPs) and false negatives (FNs). Recall was calculated as the ratio of the number of entities that were identified by the pipeline over the total number of the corresponding entities in the manually annotated gold standard, such as true positive (TP)/(TP + FN). Precision was measured as the ratio of the number of distinct entities returned by the pipeline that was correct according to the gold standard divided by the total number of entities found by our pipeline, such as TP/(TP + FP). The *F*_1_-score was calculated as the harmonic mean of PPV and sensitivity, such as 2 × PPV × sensitivity/(PPV + sensitivity). The manual annotation and training process was repeated with additional manually annotated notes until the model achieved an average *F*_1_-score >0.8.

### Characterization of GGO Cohorts and Longitudinal Analysis of GGOs

To demonstrate the utility of our GGO NLP pipeline, the NLP was deployed to the lung cancer cohort identified in the Mount Sanai/Sema4 data set to identify a cohort of patients with GGOs. Since the persistence of GGOs is an important indicator of malignancy [18,19], a subset of patients with persistent GGOs was identified by the NLP. Persistence was defined as either patients having multiple GGO reports, except when the last report indicated resolution of the GGO, or patients having only 1 GGO report but with an indication of the increase in the size or quantity or change in solidity. We used the NLP pipeline to identify GGO features from patient notes over time and assessed longitudinal changes in GGO features for this cohort.

To evaluate whether our automatically extracted information was consistent with published findings, such as larger baseline size or upper lobe location of GGOs being highly associated with the malignancy [22], we selected patients who had their first GGO report before lung cancer diagnosis date and performed a descriptive statistical analysis across the natural history of GGOs.

Finally, we extracted patients’ demographics and other clinical characteristics including smoking status, comorbidities, and family disease history from structured EHR data to characterize the population with GGOs. All statistical analyses were conducted using R software (R Foundation for Statistical Computing) and done both at the GGO level and patient level depending on the type of assessment.

## Results

### Patient Characteristics

The distribution of demographic and other clinical characteristics (ie, smoking status, comorbidities, and family history of cancer for the overall GGO cohort) over GGO persistency is shown in Table 1. The average age of the GGO cohort was 68 years; 53.77% (2431/4521) were female, and 52.95% (2394/4521) were White. Smoking data were not available for half the cohort, while among those for whom smoking data were available, 37.63% (1701/4521) of patients were either former or current smokers. Almost 70% (3086/4521) of patients had a history of cancer, and around 13% (606/4521) had a history of chronic obstructive pulmonary disease. The majority (3269/4521, 72.30%) of the GGO cohort had persistent GGOs and similar distributions of patient characteristics as the overall GGO cohort. Most GGO reports were found in the postlung cancer diagnosis period (2815/4251, 62.3%) (Figure S1 in Multimedia Appendix 1).

**Table 1:** Distribution of demographic and other clinical characterization of GGO^a^ cohorts.

Variables		Overall (N=4521), n (%)	GGO cohort persistency	
			Persistent GGO (n=3269), n (%)	Nonpersistent GGO (n=1252), n (%)
**Gender**				
	Female	2431 (53.77)	1790 (54.76)	641 (51.20)
	Male	2090 (46.23)	1479 (45.24)	611 (48.80)
**Race**				
	White	2394 (52.95)	1700 (52)	694 (55.43)
	Other	791 (17.50)	603 (18.45)	188 (15.02)
	Black or African American	722 (15.97)	530 (16.21)	192 (15.34)
	Unknown	363 (8.03)	244 (7.46)	119 (9.50)
	Asian	165 (3.65)	139 (4.25)	26 (2.08)
	Native Hawaiian or other Pacific Islander	83 (1.84)	50 (1.53)	33 (2.64)
	American Indian or Alaska Native	3 (0.07)	3 (0.09)	0 (0)
**Ethnicity**				
	Not Hispanic or Latino	2442 (54.01)	1864 (57.02)	578 (46.17)
	Unknown	1492 (33)	955 (29.21)	537 (42.89)
	Hispanic or Latino	519 (11.48)	399 (12.21)	120 (9.58)
	Not reported	68 (1.50)	51 (1.56)	17 (1.36)
**Smoking status**				
	No record of smoking	2304 (50.96)	1557 (47.63)	747 (59.66)
	Former smoker	1287 (28.47)	996 (30.47)	291 (23.24)
	Never smoker	511 (11.30)	395 (12.08)	116 (9.27)
	Smoker	414 (9.16)	317 (9.70)	97 (7.75)
	Passive smoker	5 (0.11)	4 (0.12)	1 (0.08)
**Comorbidities^b^**				
	History of COPD^c^	604 (13.36)	444 (13.58)	160 (12.78)
	History of heart disease	1297 (28.69)	924 (28.27)	373 (29.79)
	History of chronic kidney disease	340 (7.52)	262 (8.01)	78 (6.23)
	History of NMSC^d^	36 (0.80)	27 (0.83)	9 (0.72)
	History of any cancer except NMSC	3086 (68.26)	2189 (66.96)	897 (71.65)
**Family history**				
	Family history of lung cancer	8 (0.18)	7 (0.21)	1 (0.08)
	Family history of any cancer	79 (1.75)	63 (1.93)	16 (1.28)

^a^GGO: ground-glass opacity.

^b^Each patient can have more than 1 comorbidity.

^c^COPD: chronic obstructive pulmonary disease.

^d^NMSC: nonmelanoma skin cancer.

### Performance of the GGO NLP Pipeline

Among the cohort of 13,216 patients with lung cancer, 4521 (34.2%) had GGO reports, which comprised the “GGO cohort.” The NLP identified GGO features in 29,496 radiology notes of 4521 patients. Performance metrics for each GGO feature are shown in Table 2. The NLP pipeline achieved between 95% and 100% precision scores, 89% and 100% recall scores, and 92% and 100% *F*_1_-scores on different GGO features in the independent validation set. As an example, the GGO NLP algorithm correctly identified 986 pure GGOs out of 987 in the gold standard and 145 part-solid GGOs out of 146 in the gold standard with a recall of 99.7% and 99%, respectively.

**Table 2:** Quality metrics of the NLP^a^ pipeline.

Semantic	Right^b^	Predict^c^	Gold^d^	Precision	Recall	*F*_1_-score
GGO^e^ term: pure GGO	986	987	989	0.99	1	0.99
GGO term: part-solid GGO	145	146	146	0.99	0.99	0.99
GGO solidity	99	99	100	1	0.99	0.99
GGO shape/margin	144	151	144	0.95	1	0.98
GGO size	653	659	667	0.99	0.98	0.98
GGO quantity	154	156	160	0.99	0.96	0.97
GGO status change: better	46	46	46	1	1	1
GGO status change: worsen	107	107	110	1	0.97	0.99
GGO status change: stable	510	535	572	0.95	0.89	0.92
Potential GGO cause: infectious/inflammatory	146	147	148	0.99	0.99	0.99
Potential GGO cause: neoplasm	121	122	132	0.99	0.92	0.95
Potential GGO cause: others	71	73	76	0.97	0.95	0.95
GGO location	1164	1220	1270	0.95	0.92	0.93
Temporal	1650	1700	1650	0.97	1	0.99

^a^NLP: natural language processing.

^b^The number of accurately extracted entities based on the gold standard.

^c^The number of entities predicted from the NLP pipeline.

^d^Manually annotated entity by annotators.

^e^GGO: ground-glass opacity.

### GGO Characteristics

Almost all patients (n=4432, 98%) had at least 1 pure GGO in their reports, and 11% (n=505) patients had terms related to part-solid GGOs. As shown in Table 3, GGO location (3588/4521, 79.36%) was most often mentioned in notes and captured by NLP followed by potential etiology, GGO size, and change in GGO status. Over 60% (2277/3588, 63.46%) of patients had GGOs in both lungs, followed by the right lung only, with 43.42% (3948/9093 GGOs) of GGOs located in the upper lobes (Table S1 in Multimedia Appendix 1). Similarly, 43.80% (1095/2500) of patients had more than 1 potential etiology mentioned in their clinical notes, with the most common etiology being infectious or inflammatory. Around 10% (31/319) of patients in the malignant neoplasm etiology group had precancerous conditions. Among the 2350 patients identified with data on GGO size, almost half of the patients had GGOs baseline size in the range category between 6 and 20 mm (1138/2350, 48.43%), followed by >20 mm (340/2350, 14.5%) and <6 mm (274/2350, 11.6%) categories. The vast majority (845/1043, 81%) of patients with reported GGO shape or margin indicated nodules with irregular or spiculated shape, and most patients seemed to have multiple GGOs (898/904, 99.3%) rather than single GGOs (6/904, 0.7%), but data for this attribute were not frequently captured in notes. The quantity entities, even when captured, were not described as integer values in most cases but as concept values such as numerous, scattered, and several.

**Table 3:** Distribution of NLP^a^-identified GGO^b^ features in patients with GGO findings.

GGO attributes		Patients (N=4521), n (%)
Pure GGO		4432 (98)
Part solid GGO		505 (11)
**Location^c^**		
	Bilateral/both	2277 (63.5)
	Left	438 (12.2)
	Right	831 (23.2)
	Unknown/subpleural	42 (1.1)
**Potential etiology^c^**		
	Infectious/inflammatory	795 (31.8)
	Malignant neoplasm	319 (12.8)
	Other	291 (11.6)
	More than 1 cause	1095 (43.8)
**Size^c^**		
	<6 mm	274 (11.6)
	6-20 mm	1139 (48.5)
	>20 mm	340 (14.5)
	More than 1 size	597 (25.4)
**GGO status^c^**		
	Better	97 (4.2)
	Stable	1388 (59.4)
	Worse	288 (12.3)
	More than 1 status	564 (24.1)
**Shape/margin^c^**		
	Irregular/spiculated	845 (81)
	Rounded/smooth	63 (6)
	More than 1 shape	135 (13)
**Change in GGO size^d^**		
	Increase in size	240 (16.8)
	Decrease in size	208 (14.6)
	Stable in size	976 (68.5)
**Change in GGO status^e^**		
	Increased	259 (23)
	Decreased	27 (2.4)
	Stayed stable	815 (72.3)
	Resolved	26 (2.3)

^a^NLP: natural language processing.

^b^GGO: ground-glass opacity.

^c^Patient numbers were calculated from the first notes. GGO status was based on the description in the notes.

^d^Longitudinal analysis between the first and the last notes.

^e^Longitudinal analyses between the first and the subsequent notes.

### Longitudinal Analysis

Longitudinal analysis in patients with at least 2 GGO notes revealed that size increased in 16.8% (240/1424) of patients, decreased in 14.6% (208/1424), and remained unchanged in 68.5% (976/1424) in their last note compared to the first note (see Table 3 and Table S2 in Multimedia Appendix 1). The Figure S2 boxplot in Multimedia Appendix 1 shows GGO sizes at baseline and latest notes. Patients with GGO size available for only a single date were excluded from the plot. The largest GGO size was used if there was more than 1 size reported on the same day. The median GGO sizes among all relevant patients were smaller at the end point. We noticed that the patients starting with a large (>20 mm) baseline GGO size had a more medium/small GGO size reported at the end point compared with patients starting with a medium-sized GGO (see the bottom right corner split by the red lines in Figure S2 in Multimedia Appendix 1).

**Figure 4:**
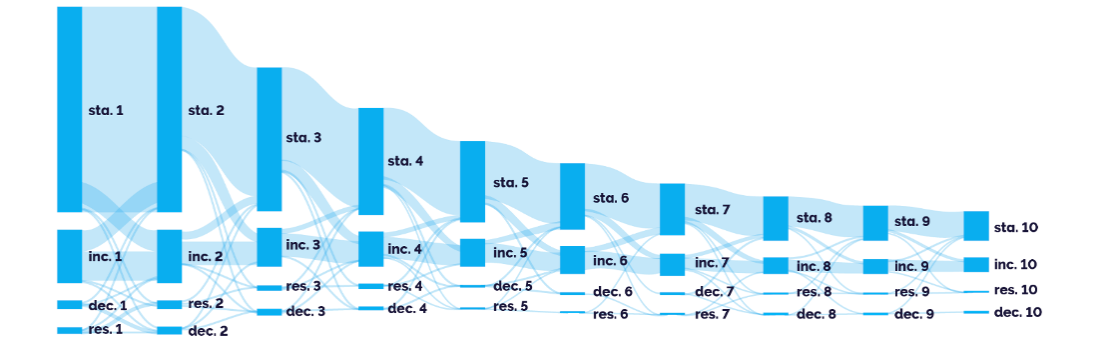
Analysis of ground-glass opacity (GGO) change in longitudinal notes. GGO status change (size and/or solidity) in the first 10 notes is visualized in the Sankey diagram. If a report had multiple status changes, the worst status change was selected. The majority of GGO stayed stable. Dec: decreased; Inc: increased; Res: resolved; Sta: stable.

A similar longitudinal analysis was performed to assess changes in GGOs over time, including indications in notes about changes in size and/or solidity or any descriptions of change. For this analysis, patients with more than 2 notes were included, and the most severe status change with the order of increased>stable>decrease was selected if more than 1 status change was reported in a day. Most patients (815/1127, 72.3%) had notes reporting a stable status of their GGOs, and “stable” was the only status reported for 40% (450/1127) of patients. The sequence of GGO status changes in the first 10 notes is depicted in Figure 4. For patients reported as stable, the subsequent report was usually stable again, followed by an increased status.

### Analysis of GGO Features and Interval Days Between GGO and Lung Cancer in the “Pregroup”

To examine whether our data are aligned with current knowledge about the impacts of size and location of nodules on lung malignancy, we analyzed GGOs in patients who had their first GGO reports before the lung cancer diagnosis date (called pregroup hereafter). Of 4521 patients with GGOs, 1706 (37.7%) were stratified into the pregroup. Among the 1706 pregroup patients, 853 (50%) patients had GGOs that can be classified exclusively into 1 baseline size group (<6 mm, 6-20 mm, or >20 mm). Table 4 shows the interval days between the first GGO report dates and the lung cancer diagnosis dates in each size group. We noted that 78% (136/174), 58% (319/550), and 47.3% (61/129) of patients had lung cancer diagnosis within 6 months in the >20 mm, 6 to 20 mm, and <6 mm groups, respectively. On the contrary, 16.6% (29/174), 31.5% (173/550), and 39.5% (51/129) of patients developed lung cancer after 1 year in the >20 mm, 6 to 20 mm, and <6 mm groups, respectively. Next, we investigated the location of GGOs in the pregroup. A total of 861 (50.5%) patients had a GGO location that could be classified into 1 location group (LLL, LUL, RLL, RML, or RUL). The upper lobe location was more frequently detected compared with the lower lobe location. Among the patients, 62.6% (539/861) had GGOs in the upper lobes, either RUL (336/861, 39%) or LUL (203/861, 23.6%). Moreover, 27.4% (236/861) of patients had GGOs in the lower lobes, either RLL (142/861, 16.5%) or LLL (94/861, 11%). The remaining 10% (86/861) of patients had GGOs in the middle lobe (RML).

**Table 4:** Patients in each size category at the different timelines from the first ground-glass opacity (GGO) notes to lung cancer diagnosis.

Size/timeline	<6 months, n (%)	6 months to 1 year, n (%)	1 year to 3 years, n (%)	>3 years, n (%)	Total, n (%)
<6 mm	61 (47.3)	17 (13.2)	29 (22.5)	22 (17)	129 (100)
6-20 mm	319 (58)	58 (10.5)	94 (17.1)	79 (14.4)	550 (100)
>20 mm	136 (78.2)	9 (5.2)	14 (8)	15 (8.6)	174 (100)

## Discussion

### Principal Findings

To understand the nature of GGOs in lung cancer cohorts, we constructed a GGO NLP pipeline in this study. Our data demonstrated high accuracy and efficiency of GGO feature identification for both pure GGOs and part-solid GGOs when this information was captured in notes. By implementing our model, we achieved automated extraction and analysis of GGO features in a huge volume of clinical notes, which enabled the identification of patients with GGOs for whom other clinical data were also available. Our model also enabled analysis of changes in GGO features over time by leveraging available longitudinal data at scale.

Similar to findings from Zheng et al [4] that utilized data from the community practices, we found that the laterality of the GGO nodules was more frequently documented in notes than other features like margins and shape. Hence, our study further supports the need for potentially standardizing the documentation of CT findings in radiology reports and progress notes. Early detection of GGOs and understanding of GGO features are critical for clinical decision-making, and they enable earlier intervention [51]. GGO status changes, including increased size and solidity, were described as critical factors for making a clinical decision on the resection [22]. Although a decrease in average nodule size has been observed across chest CT reports in general over time [4], in our study, we were able to use longitudinal data to track nodule changes specifically in each patient over time. Further analysis of whether this finding is related to treating larger GGOs can provide a better interpretation of this result and insights into GGO treatment. In our study, we also observed that the majority of patients with a GGO larger than 20 mm were diagnosed with lung cancer in the 6 months following the GGO finding.

Although GGO solidity information is one of the most critical prognostic factors [52], except for the pure or part-solid GGO information, additional GGO solidity information—such as absolute solid component sizes or solidity status changes—was not automatically extracted in previous NLP studies. In this study, we showed the feasibility of tracking the solidity status changes, as captured in the notes, but changes in every nodule may not be reflected. The solidity status changes including density change were curated by comparing the baseline and last note GGO terms. Our data revealed that most patients with solidity change information showed either a solidity increase (from pure to part solid) or stayed stable.

The quantity of GGO nodules is another crucial piece of information. It has been found that 1 to 4 GGO in a single note can be cancerous with no significant difference between 1 to 4 nodules, but ≥5 is more likely infectious/inflammatory in the etiology [53,54]. In many notes, the entities indicating the total number of GGO were not found. Radiologists described the number of GGO nodules as concepts like numerous or scattered rather than giving the actual number of GGO nodules when there are multiple GGO. Although we classified the number of GGO as multiple or single in this study, further subtyping the number of GGO nodules as 1 to 4 or ≥5 in future work by counting each GGO term extracted and their related attributes, such as location and size, could provide better insights.

### Strengths and Limitations

Although NLP technologies have significantly impacted real-world evidence generation, there remain unmet needs in clinical data retrieval such as relation recognition, longitudinal analysis, and providing insights rather than extracting data only, as Sheikhalishahi et al [39] described in their systematic review. In our deep learning model, we showed the feasibility of relation extraction rather than isolated entity extraction only and the temporal reasoning for the longitudinal analysis of patient-level data analysis. Transformer models such as BERT-based models can be examined together in future work.

There are several limitations to our study. We analyzed the GGO data in a lung cancer cohort for the initial feasibility assessment. However, our NLP pipeline can be easily expanded to other cohorts such as non–lung cancer cohorts with GGO reports in future studies, which provides more opportunities such as analyzing the associated risk factors of developing lung cancer from GGO. Additionally, a deeper analysis of pre- and postdiagnosis patient journeys can provide more insights into preventing and detecting lung malignancy. In radiology reports with multiple GGOs, tracking individual GGOs across reports over time for the longitudinal analysis of individual GGOs is challenging. Further efforts for identifying and monitoring each GGO can give us better insights into each GGO’s nature and outcome. NLP is naturally limited by its ability to capture only documented information. However, Zheng et al [4] reported trends of increasing documentation of smaller nodules and their features in radiology reports. Given this fact, NLP can be utilized as a powerful tool to study the natural history of GGOs and identify cohorts of interest for further analysis or for more in-depth radiomics work.

### Conclusions

Our study demonstrates that the deep NLP model can automatically extract granular GGO features, when documented, at scale. The model could be deployed further to large volumes of longitudinal free-text reports to continuously update prognosis as an individual’s disease course unfolds and leverage the longitudinal data with treatment patterns, clinical outcomes, and risk factors for various applications. The AI-enabled model offers a potential advantage as an automated clinical decision support tool to identify cohorts of interest for radiomics and optimize resource utilization for cancer prevention, early detection, and effective management.

## Appendix

Multimedia Appendix 1 Additional figures and tables showing duration distribution between ground-glass opacity (GGO) reports and lung cancer, analytics output of GGO size change, GGO location distribution, and longitudinal analysis of GGO size changes.

## Abbreviations

AI: artificial intelligence
BERT: Bidirectional Encoder Representations From Transformers
Bi-LSTM: bidirectional long-short term memory
CLAMP: Clinical Language Annotation, Modeling, And Processing
CRF: conditional random field
CT: computed tomography
EHR: electronic health record
FN: false negative
FP: false positive
GGO: ground-glass opacity
ICD: International Classification of Diseases
LLL: left lower lobe
LUL: left upper lobe
NLP: natural language processing
PPV: positive predictive value
RLL: right lower lobe
RML: right middle lobe
RUL: right upper lobe
TP: true positive

## Data Availability

The data used in this study are not open access due to patient privacy, security, and Health Insurance Portability and Accountability Act (HIPAA) requirements. To enable a complete run of the code shared in this study, a minimum amount of desensitized sample data could be shared with the sharing agreement. Relevant requests should be addressed to author ZL (zongzhi.liu@sema4.com). The source code of this study is provided on the GitHub website under the search term “ground glass opacity (GGO).”
