# Supplementary material for: Detecting Ground Glass Opacity Features in Patients With Lung Cancer: Automated Extraction and Longitudinal Analysis via Deep Learning–Based Natural Language Processing
Source: JMIR AI. 2023 Jun 1;2:e44537. doi: 10.2196/44537 (PMC11041451; doi:10.2196/44537)

**Multimedia Appendix 1.** Additional figures and tables showing duration distribution between ground-glass opacity (GGO) reports and lung cancer, analytics output of GGO size change, GGO location distribution, and longitudinal analysis of GGO size changes.

| **GGO location level 1** | **GGO location level 2** | **# of patients** |
| --- | --- | --- |
| Left | Lower Lobe | 1093 |
| Left | Upper Lobe | 1831 |
| Right | Lower Lobe | 1521 |
| Right | Middle Lobe | 657 |
| Right | Upper Lobe | 2117 |
| unknown/unspecified lobe |  | 1854 |
| Total |  | 9093 |

| Baseline GGO size category | Latest GGO Size category | | |
| --- | --- | --- | --- |
|  | **<6 mm** | **6-20 mm** | **>20 mm** |
| **<6 mm** | 117 | 100 | 27 |
| **6-20 mm** | 90 | 694 | 113 |
| **>20 mm** | 25 | 93 | 165 |


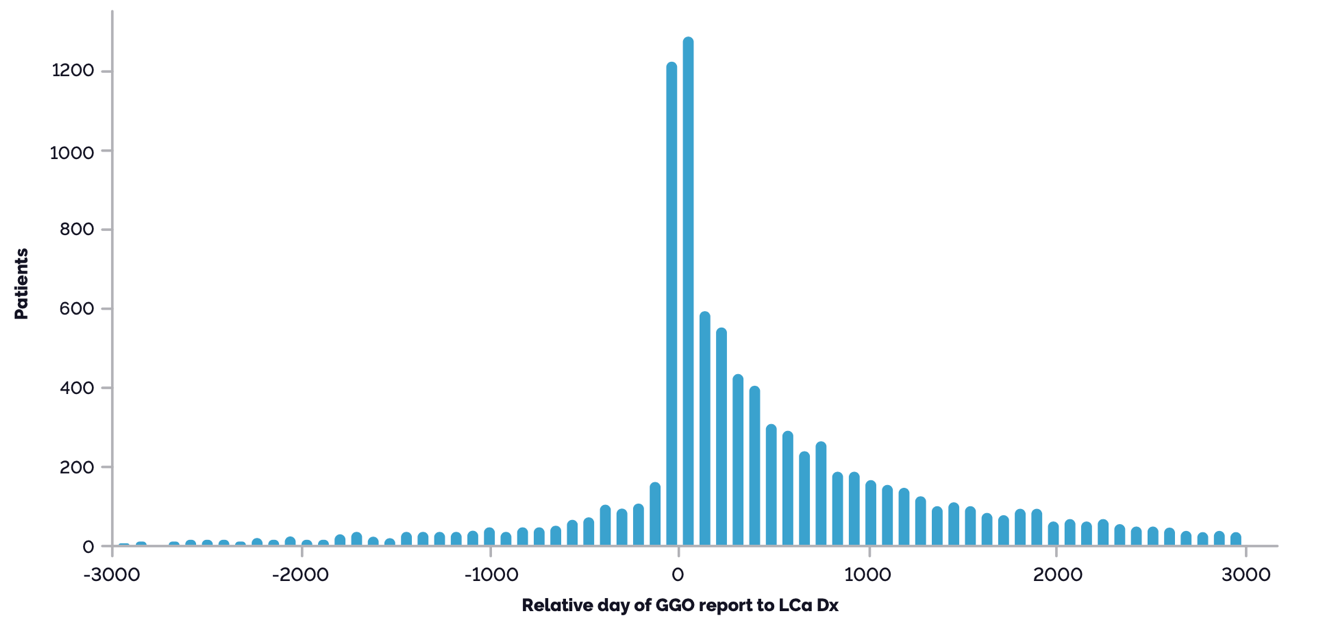


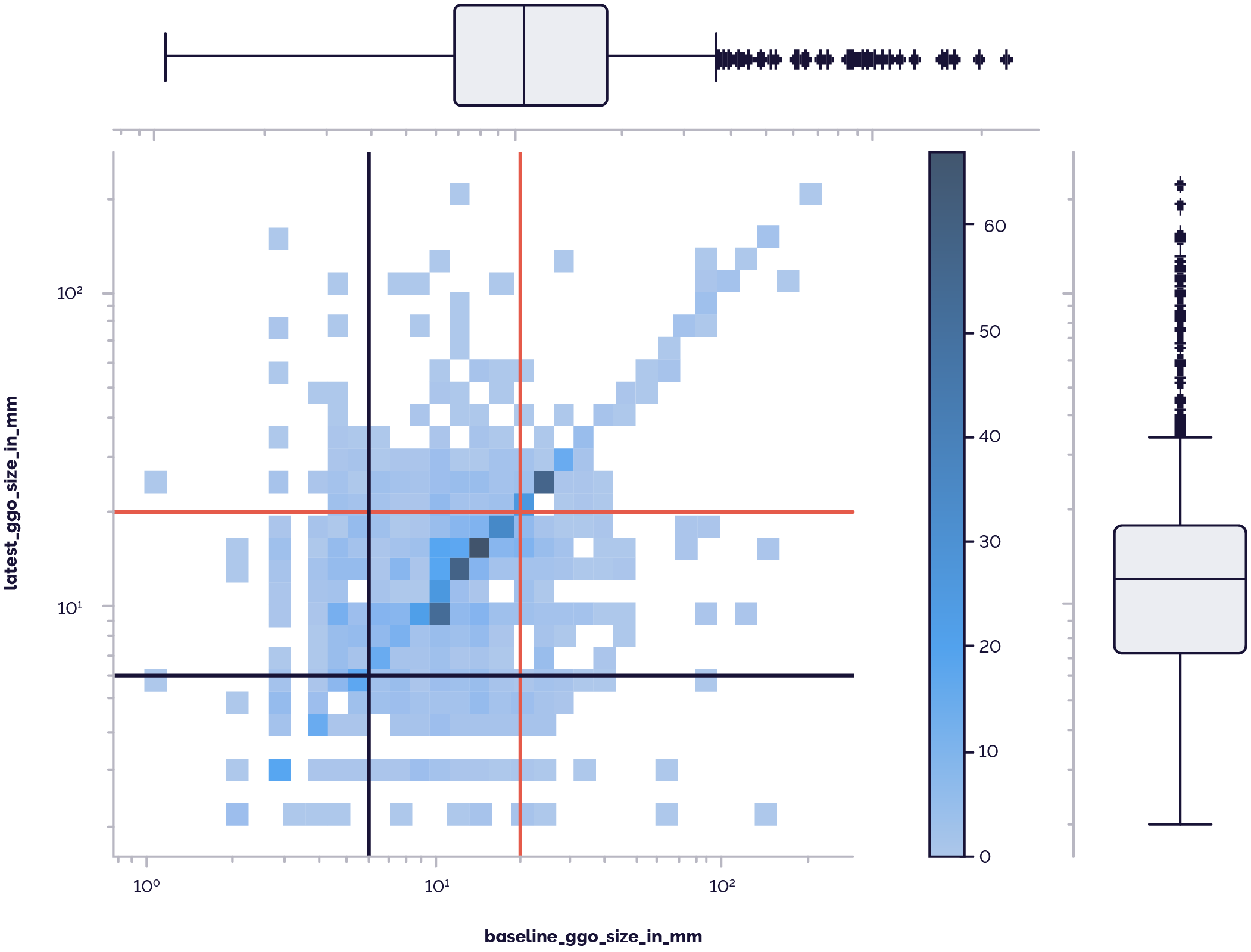

Supplement: Multimedia Appendix 1 [file ai_v2i1e44537_app1.docx]
